# Probiotic‐Enhanced Porous Bio‐Hybrids with Inflammatory Targeting, ROS Scavenging, and Long‐Term Drug Release for Ulcerative Colitis Treatment

**DOI:** 10.1002/advs.202504802

**Published:** 2025-06-27

**Authors:** Luna Quan, Yang Ouyang, Weiwen Liang, Zixin Chen, Dongtian Miao, Bingna Zheng, Dingcai Wu, Rongkang Huang

**Affiliations:** ^1^ PCFM Lab School of Chemistry Sun Yat‐sen University Guangzhou 510006 P. R. China; ^2^ Colorectal Surgery Unit III Guangdong Institute of Gastroenterology Biomedical Innovation Center Guangdong Provincial Key Laboratory of Colorectal and Pelvic Floor Diseases The Sixth Affiliated Hospital Sun Yat‐sen University Guangzhou 510655 P. R. China; ^3^ The Eighth Affiliated Hospital Sun Yat‐sen University Shenzhen 518000 P. R. China

**Keywords:** hyper‐cross‐linking polymers, inflammatory targeting, long‐term drug release, ROS scavenging, ulcerative colitis

## Abstract

Functional porous materials hold significant promise for biomedical applications owing to their high surface area and customizable pore architectures. However, the complex gastrointestinal environment poses considerable challenges for conventional nanomaterials in achieving targeted accumulation and controlled drug release. Herein, a kind of novel probiotic‐enhanced porous bio‐hybrids (E‐*x*PAM@ASA) is designed via bio‐hybridization of 5‐aminosalicylic acid‐loaded hairy microporous nanospheres (*x*PAM@ASA) with probiotic *Escherichia coli* Nissle 1917. Benefiting from the intrinsic inflammatory‐targeting capability of EcN, the E‐*x*PAM@ASA can accumulate in the inflammatory sites of the intestine. The unique porous architecture of *x*PAM@ASA not only facilitates high drug loading and long‐term release but also provides abundant adsorption sites for effective reactive oxygen species scavenging. In a dextran sulfate sodium‐induced ulcerative colitis murine model, E‐*x*PAM@ASA demonstrate superior therapeutic outcomes, including mucosal repair, inflammation alleviation, and microbiota regulation. These findings highlight the potential of the multifunctional nanocomposite as a precise therapeutic platform for the treatment of intestinal inflammation.

## Graphical abstract

Probiotic‐enhanced porous bio‐hybrids (E‐*x*PAM@ASA), developed by coupling the drug‐loaded hairy microporous nanospheres *x*PAM@ASA with probiotic EcN, integrate inflammatory‐targeting, ROS scavenging, and long‐term release of 5‐ASA to improve inflammation and regulate microbiota structure in the DSS‐induced colitis model, providing an advanced strategy for the precise treatment of ulcerative colitis.

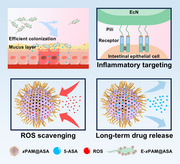

## 1 Introduction

Porous nanomaterials have attracted great interest in biomedical applications, particularly in anti‐inflammatory and anti‐cancer therapies, owing to their high specific surface area, customizable pore architecture, and exceptional drug‐loading capacity.^[^ ^1^, ^2^, ^3^ ^]^ These structural advantages position them as promising candidates for addressing complex inflammatory pathologies, particularly ulcerative colitis (UC), a prevalent type of inflammatory disease characterized by compromised gut epithelial barrier integrity and microbial dysbiosis.^[^ ^4^ ^]^ The pathogenesis of UC involves excessive immune activation marked by elevated levels of reactive oxygen species (ROS) and pro‐inflammatory mediators.^[^ ^5^, ^6^ ^]^ The abundant adsorption sites and surface activity of porous nanomaterials enable their efficient scavenging of ROS, while their well‐developed pore structures facilitate targeted delivery of therapeutic agents to restore mucosal barrier function.^[^ ^7^, ^8^ ^]^ However, the dynamic complexity of the gastrointestinal environment, including pH fluctuations, peristaltic motility, and mucosal barrier resistance, poses significant challenges for conventional porous nanomaterials to achieve targeted accumulation in the inflammatory sites of the intestine.^[^ ^9^, ^10^ ^]^ In addition, these materials often exhibit non‐specific diffusion or premature payload leakage of bioactive agents before reaching the target sites.^[^ ^11^ ^]^ Therefore, developing novel porous nanocomposites that synergistically integrate inflammatory‐targeting capability, ROS scavenging functionality, and controlled drug release properties through various functional modifications has emerged as a crucial strategy to address the therapeutic bottlenecks of UC.

The functional modifications of porous nanomaterials pose inherent challenges in balancing the retention of porous structures and the enhancement of therapeutic efficacy.^[^ ^12^ ^]^ For example, the application of mucoadhesive polymer coatings can enhance intestinal targeting efficiency through ligand‐receptor interactions.^[^ ^13^, ^14^ ^]^ However, these strategies often compromise the materials’ intrinsic porous structure due to pore occlusion (e.g., 84% reduction of specific surface area after modification)^[^ ^15^ ^]^ or active site masking, leading to insufficient utilization of the well‐developed pores.^[^ ^16^, ^17^ ^]^ In another example, hydrogel‐encapsulated porous nanocomposite systems demonstrate improved lesion retention through physical entrapment, but their viscoelastic matrices could alter the interfacial interactions between the functional porous frameworks of nanomaterials and the intestinal mucosa.^[^ ^18^ ^]^ Moreover, most current modification strategies focus on a single functional improvement, which makes it challenging to address UC's multifactorial pathogenesis, thus requiring concurrent targeting, sustained drug release, and microenvironment regulation.^[^ ^19^ ^]^ This predicament has spurred interest in probiotic‐driven solutions, which offer dual advantages over conventional antibody/ligand‐based targeting methods.^[^ ^20^, ^21^ ^]^ For example, the intrinsic inflammatory chemotaxis of *Escherichia coli* Nissle 1917 (EcN) overcomes the off‐target risks of traditional ligands, while its bioactivity synergistically regulates the lesion microenvironment.^[^ ^22^ ^]^ Therefore, the construction of multifunctional EcN‐integrated nanocomposites with inflammatory targeting, long‐term drug release, and microenvironment regulation properties represents a transformative approach to UC therapy.

**Figure 1:**
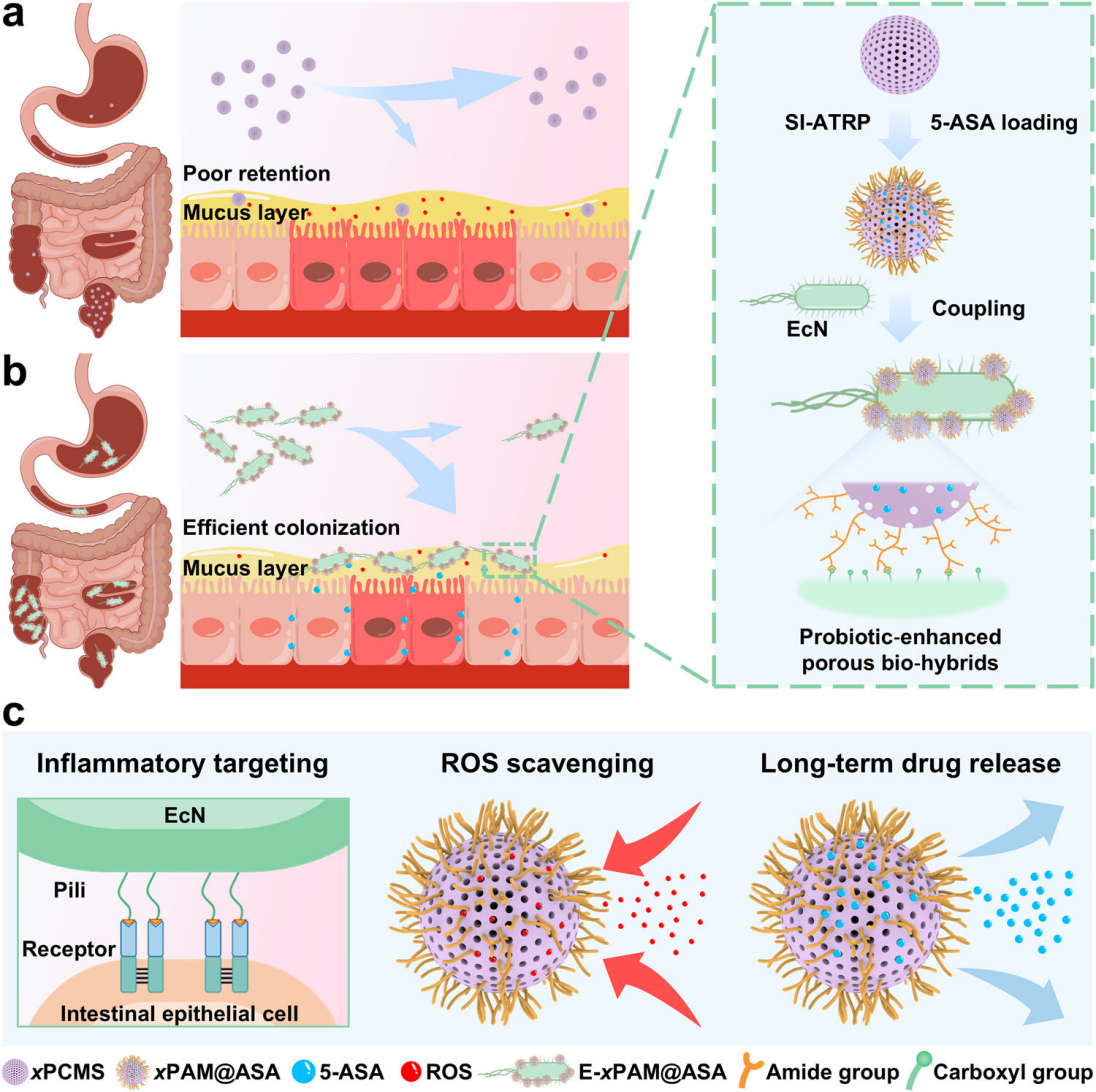
a, b) Schematic diagram of intestinal retention capability of classical porous nanomaterials compared to our probiotic‐enhanced porous bio‐hybrids E‐*x*PAM@ASA: a) Classical porous nanomaterials lack bioactivity and fail to colonize the inflamed regions of the intestine, leading to rapid clearance and limited therapeutic effects. b) E‐*x*PAM@ASA, benefiting from probiotic‐assisted targeting, achieves effective retention at inflamed sites, enabling ROS scavenging and sustained drug release for inflammation recovery. Synthesis process of E‐*x*PAM@ASA involves the surface‐functionalization of *x*PCMS by SI‐ATRP to form hairy microporous polymer nanospheres *x*PAM, followed by the loading of 5‐ASA and subsequent coupling with EcN. c) Key functional attributes of E‐*x*PAM@ASA: inflammatory‐targeting through EcN‐assisted homing, ROS scavenging facilitated by the microporous structure, and long‐term release of 5‐ASA for sustained therapeutic effects.

Herein, we have successfully developed a kind of novel probiotic‐enhanced porous bio‐hybrids with excellent inflammatory targeting capacity, ROS scavenging ability, and long‐term drug release performance using a facile chemical modification approach (**Figure** **1**). Specifically, polyacrylamide (PAM) side‐chains are grafted from microporous nanospheres (*x*PCMS) via surface‐initiated atom transfer radical polymerization (SI‐ATRP), obtaining the hairy microporous polymer nanospheres (*x*PCMS‐*g*‐PAM, denoted as *x*PAM). After loading anti‐inflammatory drug 5‐aminosalicylic acid (5‐ASA) through a simple physical adsorption,^[^ ^23^ ^]^ the amino groups of drug‐loaded *x*PAM (*x*PAM@ASA) are connected with the carboxyl groups on the surface of probiotic EcN by a coupling reaction,^[^ ^24^ ^]^ finally obtaining the probiotic‐enhanced porous bio‐hybrids (E‐*x*PAM@ASA). The intrinsic inflammation‐homing capability of EcN enables the targeted enrichment of E‐*x*PAM@ASA, thereby significantly enhancing the retention of E‐*x*PAM@ASA within intestinal lesions. Meanwhile, the well‐developed porous structure of *x*PAM@ASA enables E‐*x*PAM@ASA to effectively scavenge ROS and sustainedly release 5‐ASA, thereby regulating the microenvironment of the inflammatory sites. Benifitting from the above properties, our multifunctional bio‐hybrids demonstrate effective promotion of tissue repair and regulation of microenvironment in a dextran sulfate sodium (DSS)‐induced UC murine model. The concept and results presented in our work could offer an advanced strategy for the precise delivery of nanomaterials and treatment of intestinal diseases.

## 2 Results and Discussion

### 2.1 Preparation and Structure Characterization

**Figure 2:**
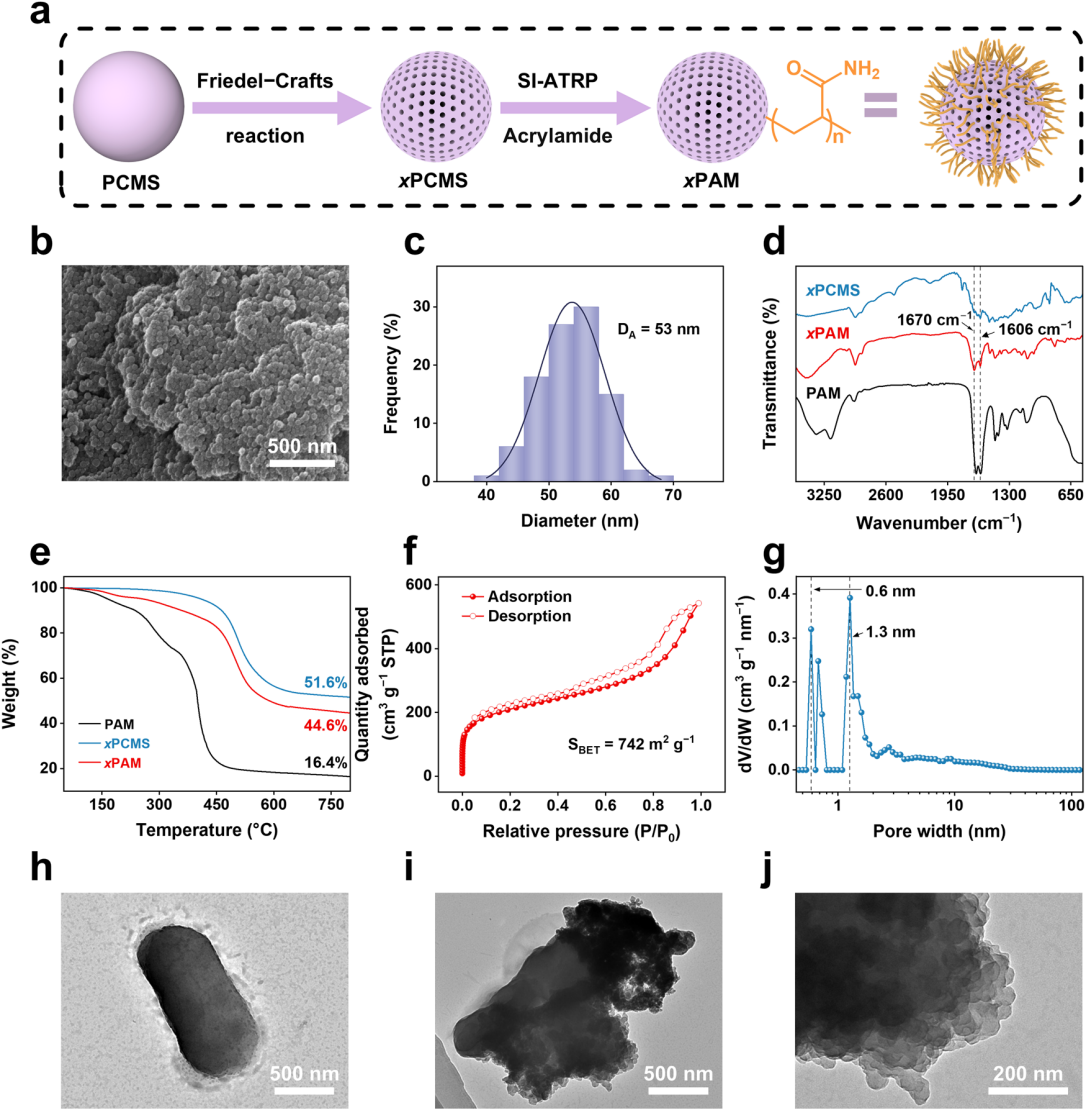
a) Schematic illustration of the preparation of *x*PAM. b) Scanning electron microscopy (SEM) image of *x*PAM. c) Particle size distribution curve of *x*PAM according to the SEM image. d) FTIR spectra of PAM, *x*PCMS, and *x*PAM. e) TGA curves of PAM, *x*PCMS, and *x*PAM. f, g) N_2_ adsorption‐desorption isotherm at 77 K (f) and density functional theory pore size distribution curve (g) of *x*PAM. h–j) TEM images of EcN (h), E‐*x*PAM (i), and *x*PAM within E‐*x*PAM (j).

The preparation procedures of functional porous nanospheres include emulsion polymerization, Friedel–Crafts hyper‐cross‐linking reaction, and SI‐ATRP. Initially, poly(4‐chloromethylstyrene) nanospheres with divinylbenzene pre‐cross‐linking (PCMS) were prepared by emulsion polymerization, which endows PCMS with uniform nanospherical nanomorphology. Subsequently, microporous polymeric nanospheres *x*PCMS with permanent microporous structure were obtained by Friedel–Crafts hyper‐cross‐linking reaction.^[^ ^25^ ^]^ The residual benzyl chloride groups in *x*PCMS can act as initiating sites to trigger SI‐ATRP of acrylamide, thus obtaining hairy microporous nanospheres *x*PAM grafted by polyacrylamide that can be used as the multifunctional component (**Figure** **2**a).

As shown in Figure 2b,c and Figure S1 (Supporting Information), *x*PAM can retain the nanospherical morphology of *x*PCMS after SI‐ATRP, with an average diameter of 53 nm. The fourier‐transform infrared (FTIR) spectrum of *x*PAM shows new absorption peaks at 1670 and 1606 cm⁻¹, corresponding to the amide I band (C═O stretching vibration) and amide II band (N─H stretching vibration), respectively, confirming the successful grafting of PAM (Figure 2d).^[^ ^26^ ^]^ Thermogravimetric analysis (TGA) measurements show that *x*PAM display a weight‐loss stage compared with *x*PCMS, further confirming the successful modification of organic functional groups and hairy PAM chains, with a calculated PAM content of 18 wt% (Figure 2e). The PAM side‐chains can enhance the dispersibility of *x*PAM in water (Figure S2, Supporting Information), laying the foundation for subsequent bio‐hybridization. The porous structure of *x*PAM was characterized by the nitrogen adsorption method. The calculated Brunauer‐Emmett‐Teller surface area (S_BET_) of *x*PAM is 742 m^2^ g^−1^, with pore sizes mainly distributed at 0.6 and 1.3 nm (Figure 2f,g), retaining the well‐developed microporous structure of *x*PCMS (1093 m^2^ g^−1^, Figure S3, Supporting Information). This microporous structure is critical for loading drugs and scavenging ROS.

Subsequently, the PAM side‐chains were successfully attached to the carboxyl‐rich EcN via 1‐ethyl‐3‐(3‐dimethylaminopropyl) carbodiimide/N‐hydroxysuccinimide coupling reaction. Transmission electron microscopy (TEM) analysis (Figure 2h–j; Figure S4, Supporting Information) demonstrates that within the E‐*x*PAM composite, EcN retains its characteristic rod‐shaped morphology while *x*PAM preserve their nanospherical structure. It indicates that the coupling process does not affect the morphology of probiotic or *x*PAM. As the presence of carboxyl groups in the surface proteins and extracellular polysaccharides of probiotic surfaces is a common feature of various probiotics, this coupling reaction offers a versatile and generalizable platform to hybridize our *x*PAM with a wide range of probiotics.

### 2.2 Drug Release and ROS Scavenging

**Figure 3:**
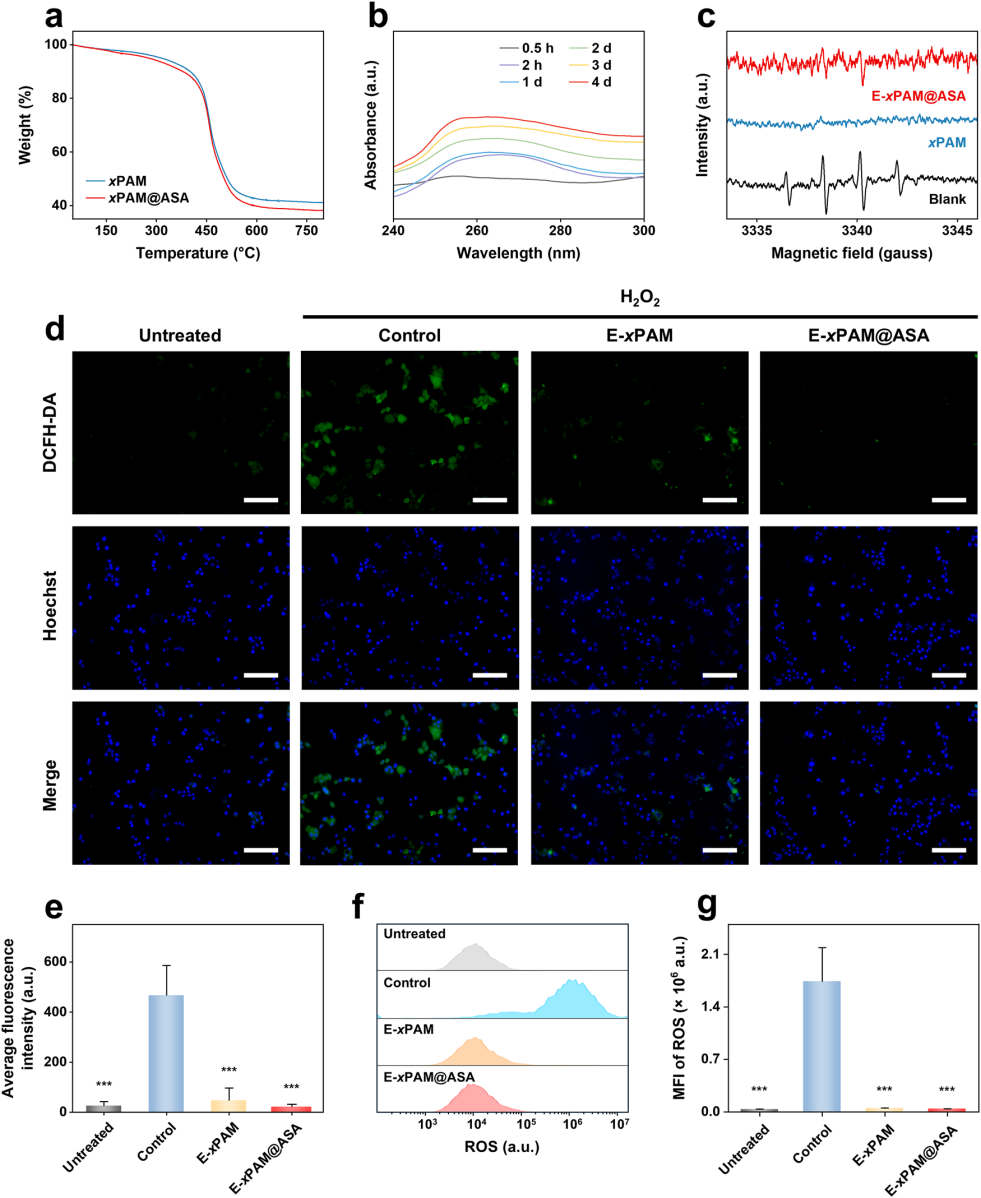
a) TGA curves of *x*PAM and *x*PAM@ASA. b) Time‐dependent release of 5‐ASA from *x*PAM@ASA in PBS. c) EPR spectra of DMPO‐•OH in the presence of different samples and TiO_2_ under UV irradiation. d) Fluorescence images and e) corresponding fluorescence intensities of NCM460 cells with different treatments. The cells were stained with DCFH‐DA (green fluorescence) and Hoechst (blue fluorescence). f) Histograms of ROS expression of NCM460 cells and g) the MFI of ROS in NCM460 cells with different treatments. Scale bars: 50 µm (d). The data are the mean ± standard deviation (SD) (n = 3 independent samples; ^***^ *p* < 0.001).

To evaluate the drug loading capacity of *x*PAM, 5‐ASA is loaded into *x*PAM to obtain drug‐loaded porous nanospheres *x*PAM@ASA with a loading amount of 70 mg g^−1^ calculated by TGA curves (**Figure** **3**a). Subsequently, PBS‐dispersed *x*PAM@ASA was oscillated at 37 °C, and the drug release solution was collected to assess the drug release profile. The UV absorption spectra at different time points show that *x*PAM@ASA gradually release 5‐ASA within 4 days (Figure 3b). Electron paramagnetic resonance (EPR) spectroscopy was employed to verify the ROS scavenging ability of *x*PAM. As shown in Figure 3c, a strong signal of 5,5‐dimethyl‐1‐pyrroline‐N‐oxide (DMPO)‐•OH is detected in the blank group. In sharp contrast, the signal of DMPO‐•OH in the *x*PAM group is significantly weakened, demonstrating the excellent •OH scavenging capability of *x*PAM. Importantly, the E‐*x*PAM@ASA group maintains a comparable signal suppression efficiency, indicating that the bio‐hybridization of *x*PAM@ASA and EcN does not compromise the ROS scavenging performance. To further evaluate the ROS scavenging ability of E‐*x*PAM@ASA, 2,2‐diphenyl‐1‐picrylhydrazyl (DPPH), a widely used stable free radical, was employed for the scavenging assay. The UV–vis absorption intensity of DPPH after treatment with E‐*x*PAM@ASA is significantly lower than that of EcN (Figure S5, Supporting Information), further confirming that the introduction of *x*PAM@ASA endows EcN with free radical scavenging ability.^[^ ^27^ ^]^ Overall, the well‐developed microporous structure of *x*PAM provides efficient channels for loading and sustainedly releasing drugs as well as scavenging ROS.

To further validate the in vitro antioxidant capability of *x*PAM@ASA for UC treatment, an oxidative microenvironment model was established by stimulating normal human colon mucosal epithelial (NCM460) cells with hydrogen peroxide (H_2_O_2_). The intracellular ROS levels in NCM460 cells under various treatment conditions were assessed using fluorescence microscopy. Cells were dual‐stained with 2′,7′‐dichlorodihydrofluorescein diacetate (DCFH‐DA), a fluorogenic probe for ROS detection, and Hoechst 33342, a cell‐permeant nuclear counterstain. As shown in Figure 3d,e, untreated cells display physiological ROS levels, as evidenced by minimal green fluorescence intensity. In contrast, H_2_O_2_ stimulation can induce significant oxidative stress, elevating fluorescence intensities in the control group. However, the E‐*x*PAM group shows a remarkable reduction in the green fluorescence intensity and elevation in the blue fluorescence intensity, indicating the superior intracellular ROS scavenging capacity of E‐*x*PAM with a well‐developed porous architecture. Notably, the E‐*x*PAM@ASA group exhibits the lowest green fluorescence intensity among all the groups, with the loaded 5‐ASA providing additional ROS scavenging through its antioxidant and free radical‐scavenging properties. To further quantitatively assess the intracellular ROS levels, the mean fluorescence intensity (MFI) of different groups was evaluated by using flow cytometry. As shown in Figure 3f,g, the E‐*x*PAM@ASA group demonstrates a significantly lower MFI compared to other groups, indicating reduced ROS accumulation. These results confirm that E‐*x*PAM@ASA can effectively scavenge cellular ROS, thereby enhancing its therapeutic potential.

### 2.3 Probiotic Activity and Intestinal Retention

**Figure 4:**
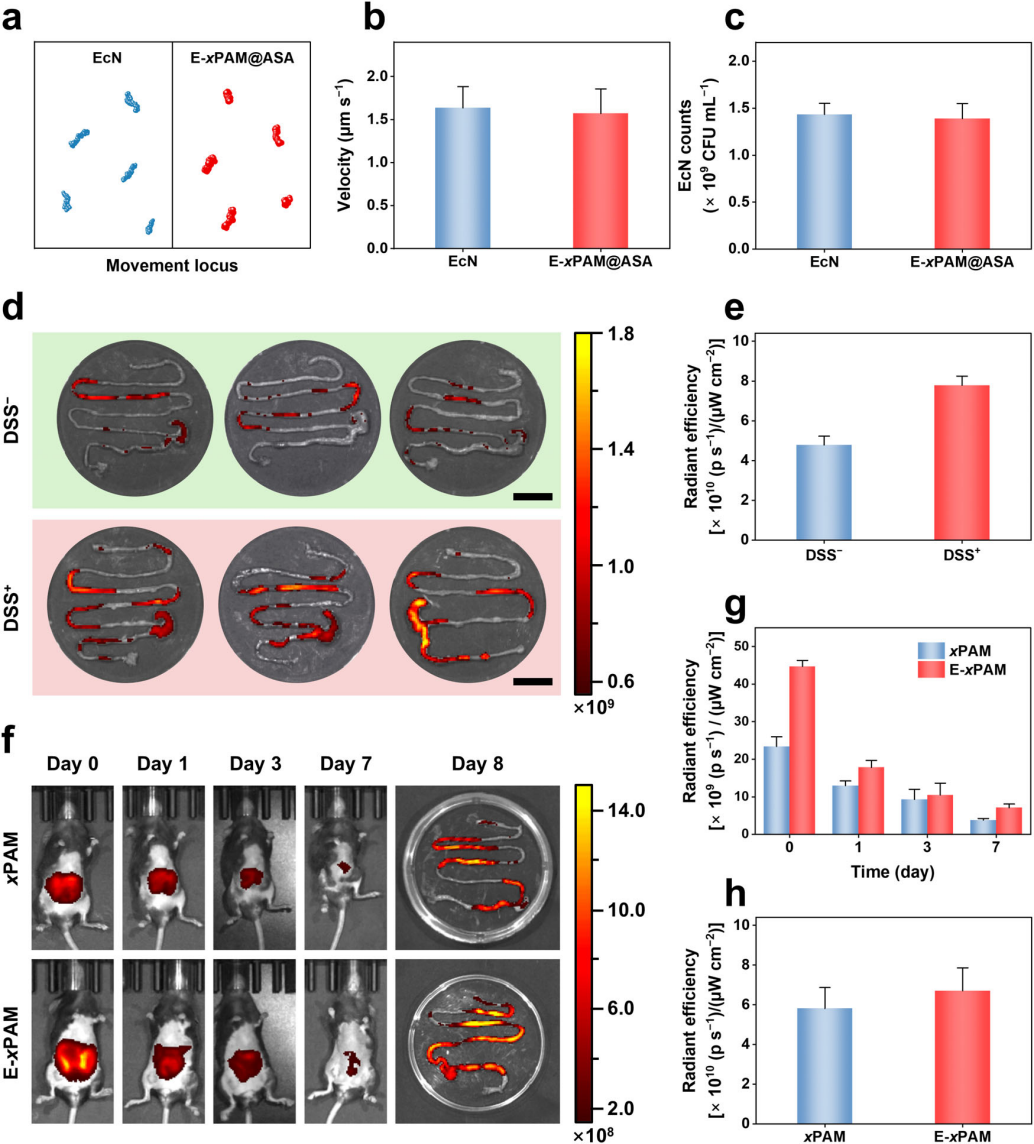
a, b) Representative trajectories (a) and average velocity (b) of EcN and E‐*x*PAM@ASA recorded within 1 min. The data are the mean ± SD (n = 50 independent samples). c) Colony count statistics of EcN and E‐*x*PAM@ASA after 8 h of incubation in LB medium. d, e) Fluorescence images of the mice intestine in the DSS^−^ and DSS^+^ groups recorded 24 h after gavage administration of Cy5.5‐labeled E‐*x*PAM (d) and the corresponding statistics related to the radiant efficiency (e). f–h) Fluorescence images (f) and the corresponding statistics related to the radiant efficiency of mice (g) and the mice intestine (h) in the *x*PAM and E‐*x*PAM groups at different times. Scale bars: 2 cm (d). The data are the mean ± SD (n = 3 independent samples).

To verify whether the incorporation of *x*PAM@ASA onto the probiotic carrier affects probiotic activity and motility in the intestinal environment, we have conducted a comparative analysis of the motility and proliferation of EcN before and after the incorporation of *x*PAM@ASA. After the coupling reaction between *x*PAM@ASA and EcN for 3 h, probiotic survival of E‐xPAM@ASA shows no significant decrease compared to natural EcN (Figure S6, Supporting Information). The probiotic suspension was diluted, and the trajectories of individual cells were observed.^[^ ^28^ ^]^ Both native EcN and E‐*x*PAM@ASA exhibit clear movement trajectories (**Figure** **4**a). The average speeds, calculated based on the total distance traversed over time, are 1.64 ± 0.25 µm s^−1^ for EcN and 1.57 ± 0.28 µm s^−1^ for E‐*x*PAM@ASA (Figure 4b). This slight reduction may be attributed to the incremental mass and hindrance of flagellar motion conferred by the drug‐loaded porous nanospheres, together with the decrease in probiotic activity after chemical reactions.^[^ ^29^, ^30^ ^]^ In addition, following the dilution of EcN and E‐*x*PAM@ASA in Luria‐Bertani (LB) medium and subsequent incubation for 8 h, plate counting was performed (Figure S7, Supporting Information). Statistical analysis shows that both groups exhibit comparable colony densities on agar plates (Figure 4c), thereby demonstrating that the functionalization process does not negatively affect the proliferation activity of probiotic. In addition, CCK‐8 and live/dead staining assays confirm that *x*PAM exhibit no significant toxicity toward colonic epithelial cells (Figure S8, Supporting Information).

To validate the enhancing effect of EcN on the intestinal retention of *x*PAM, the in vivo imaging system (IVIS) was employed to compare the retention of Cy5.5‐labeled E‐*x*PAM in healthy mice (DSS^−^) and DSS‐induced colitis mice (DSS^+^). Twenty‐four hours after gavage of Cy5.5‐labeled E‐*x*PAM, the mice were euthanized, and their intestines were collected for fluorescence imaging. As shown in Figure 4d,e, distinct fluorescence signals are detected in both the DSS^−^ and DSS^+^ groups, with the fluorescence intensity in the intestinal regions of DSS^+^ mice being significantly higher than that in the DSS^−^ group (*p* < 0.01). This suggests the enrichment capability of E‐*x*PAM at intestinal lesions.^[^ ^31^ ^]^ To evaluate the retention dynamics of *x*PAM and E‐*x*PAM in the gut, we have tracked the fluorescence signals of Cy5.5‐labeled *x*PAM and E‐*x*PAM on Day 0, 1, 3, and 7 post‐gavage. As shown in Figure 4f, both groups exhibit strong initial fluorescence signals on Day 0, and the *x*PAM group exhibits a more rapid attenuation of fluorescence intensity. In contrast, the E‐*x*PAM group maintains a higher fluorescence signal during the test period. Quantitative analysis of the radiant efficiency in both whole‐body imaging and dissected intestinal tissues reveal that E‐*x*PAM exhibit greater fluorescence intensity compared to *x*PAM, particularly on Day 7 (Figure 4g,h). These findings demonstrate that the colonization capability of EcN endows E‐*x*PAM with prolonged retention time in inflammatory intestine.^[^ ^32^, ^33^ ^]^

### 2.4 Therapeutic Efficacy on Ulcerative Colitis

**Figure 5:**
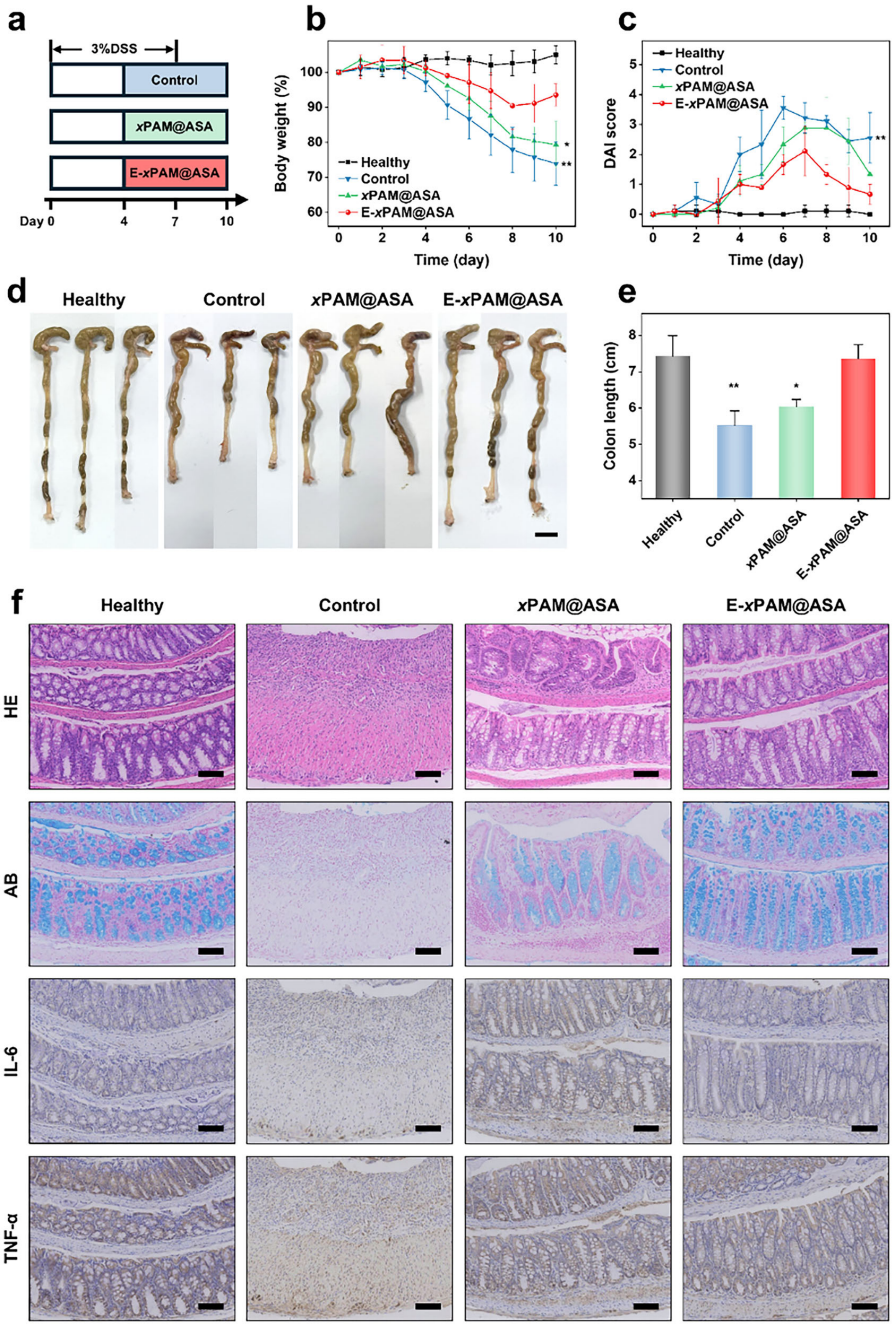
a) Schematic diagram outlining the treatment of colitis in C57BL/6 mice. b) Daily body weight changes in each group for 10 days. Data are normalized as a percentage of the body weight on Day 0. c) Changes in DAI for 10 days. d, e) Digital photos of the colons (d) and the corresponding statistics related to the colon length (e) of the mice in each group. f) Images of HE staining, AB staining, and immunohistochemical staining of IL‐6 and TNF‐α for each group. Scale bars: 1 cm (d) and 100 µm (f). The data are the mean ± SD (n = 3 independent samples; ^*^ *p* < 0.05, ^**^ *p* < 0.01).

To evaluate the therapeutic potential of E‐*x*PAM@ASA for UC treatment, an acute colitis murine model was established by administering 3% DSS in drinking water for 7 consecutive days, with therapeutic interventions initiated on Day 4 (**Figure** **5**a). The DSS‐induced mice were divided into three groups: control (PBS‐treated), *x*PAM@ASA‐treated, and E‐*x*PAM@ASA‐treated groups. In addition, a healthy group was included as the negative control without any treatment, in which mice received normal drinking water instead of DSS. Body weight variations were monitored as an indirect indicator of therapeutic efficacy. The E‐*x*PAM@ASA group exhibits superior body weight stabilization compared to both the control and *x*PAM@ASA groups (Figure 5b). Disease progression is further evaluated through the disease activity index (DAI), with the E‐*x*PAM@ASA group displaying a significantly reduced DAI score (Figure 5c; Table S1, Supporting Information). Given that UC could lead to chronic tissue damage and colon shortening, the colons of different groups were isolated, imaged, and measured to further evaluate tissue injury. Compared to the healthy group, the E‐*x*PAM@ASA group displays an average reduction of 0.9%, which is lower than those of the control (25.7%) and *x*PAM@ASA (18.8%) groups (Figure 5d,e), indicating that the therapeutic capability of E‐*x*PAM@ASA for UC treatment is enhanced in the presence of EcN.

To further validate the therapeutic efficacy of E‐*x*PAM@ASA, hematoxylin−eosin (HE) staining, alcian blue (AB) staining, and immunohistochemical staining were performed for a comprehensive analysis of tissue morphology, extracellular matrix composition, and inflammatory response, respectively. As illustrated in Figure 5f and Figure S9 (Supporting Information), HE staining of colonic lesions shows that the control group exhibits a complete loss of goblet and crypt cells, whereas the E‐*x*PAM@ASA group shows near‐physiological mucosal and mucus regeneration; AB staining shows that there is a more complete intestinal barrier in the E‐*x*PAM@ASA group compared to the control group. As shown in Figure 5f and Figure S10 (Supporting Information), immunohistochemical staining and semi‐quantitative analysis of pro‐inflammatory factors show that the expression levels of interleukin‐6 (IL‐6) and tumor necrosis factor‐alpha (TNF‐α) in the E‐xPAM@ASA group are significantly lower compared to those in the control group. Systemic biosafety assessment through HE staining of major organs (heart, liver, spleen, lungs, and kidneys) reveals no detectable pathological abnormalities following treatment (Figure S11, Supporting Information). These results indicate that our E‐*x*PAM@ASA exhibit superior ROS clearance, sustained drug release, and inflammatory‐targeting enrichment properties and thus provides an ideal approach to obtain effective treatment of UC.

### 2.5 Modulatory Effect on Gut Microbiome

**Figure 6:**
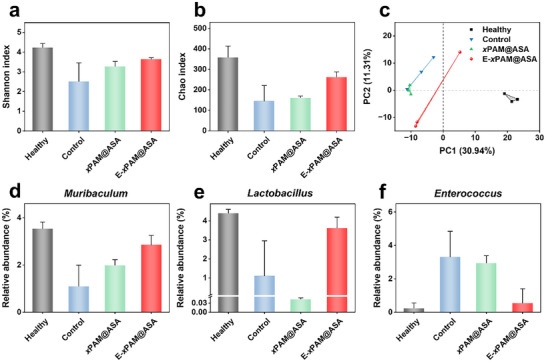
a–c) The α‐diversity analysis of fecal microbiome illustrated by Shannon's (a) and Chao's index (b), and the β‐diversity analysis (c) of fecal microbiome demonstrated by PCA plot in different treatment groups on Day 10. d–f) Relative genus abundance of *Muribaculum* (d), *Lactobacillus* (e), and *Enterococcus* (f) in different treatment groups on Day 10. The data are the mean ± SD (n = 3 independent samples).

Since gut microecology imbalance is an important characteristic of colitis, the effect of E‐*x*PAM@ASA on gut microbiome composition was investigated.^[^ ^34^ ^]^ Feces of mice in all groups were collected on Day 10 and examined by using 16S rRNA gene sequencing. The α‐diversity analyses with Shannon and Chao demonstrate that the diversity and abundance of fecal microbiome in the E‐*x*PAM@ASA group are improved compared to those in the control group, and are similar to those in the healthy group (**Figure** **6**a,b). The principal component analysis (PCA) for β‐diversity shows that the fecal microbiome of the control group is significantly distinct from the healthy group, while the E‐*x*PAM@ASA group is similar to that of the healthy group (Figure 6c). To further evaluate the regulatory effect of the E‐*x*PAM@ASA on specific fecal microbiome, the abundances of microbiome at the phylum and genus levels were quantitatively analyzed. According to the abundance of the phylum (Figure S12, Supporting Information), compared with the control group, the E‐*x*PAM@ASA group shows similar fecal microbiome characteristics to those of the healthy group, indicating a significant increase in the Bacteroidota phylum and a significant decrease in the Pseudomonadota phylum. According to the abundance of the genus, E‐*x*PAM@ASA can significantly improve the microbiome structure (Figure S13, Supporting Information). Compared with the control group, there is an increase in the relative abundances of *Muribaculum* (Figure 6d) and *Lactobacillus* (Figure 6e) in the E‐*x*PAM@ASA group, which can effectively alleviate the symptoms of colitis by enhancing the intestinal barrier and anti‐inflammatory properties.^[^ ^35^, ^36^ ^]^ In contrast, the relative abundance of *Enterococcus* (Figure 6f), which is reported to produce various virulence factors such as hemolysin and gelatinase that contribute to the progression of inflammation, is remarkably decreased in the E‐*x*PAM@ASA group.^[^ ^37^, ^38^ ^]^ Therefore, our E‐*x*PAM@ASA can increase probiotics and reduce harmful bacteria, thus reshaping the balance of the gut microecology in the colitis microenvironment.

## 3 Conclusion

In summary, we have successfully developed a novel class of probiotic‐enhanced porous bio‐hybrids by coupling the drug‐loaded hairy microporous nanospheres *x*PAM@ASA with EcN. Our E‐*x*PAM@ASA demonstrates inflammatory targeting, ROS scavenging, and long‐term drug release properties, which are benefited from the intrinsic inflammation‐homing capability of EcN carrier and the unique functional microporous architecture of *x*PAM@ASA. Such unique E‐*x*PAM@ASA bio‐hybrids can accumulate in the inflammatory sites of the intestine, thereby alleviating the inflammatory burden and improving the intestinal microenvironment. Meanwhile, E‐*x*PAM@ASA provide long‐term drug release to enhance the anti‐inflammatory treatment of UC. Consequently, our E‐*x*PAM@ASA show potential to promote intestinal mucosal repair and microbiota regulation in the DSS‐induced ulcerative colitis murine model. Our work could offer a promising direction for the development of multifunctional platforms for ulcerative colitis treatment.

## 4 Experimental Section

The Experimental Section is available in the Supporting Information.

## Conflict of Interest

The authors declare no conflict of interest.

## Supporting information



Supporting Information

## Data Availability

The data that support the findings of this study are available in the supplementary material of this article.
