# Supplementary material for: Probiotic‐Enhanced Porous Bio‐Hybrids with Inflammatory Targeting, ROS Scavenging, and Long‐Term Drug Release for Ulcerative Colitis Treatment
Source: Adv Sci (Weinh). 2025 Jun 27;12(32):e04802. doi: 10.1002/advs.202504802 (PMC12407365; doi:10.1002/advs.202504802)
Supplement: Supplementary file 1 — Supporting Information [file ADVS-12-e04802-s001.docx]

Supporting Information

**Probiotic-Enhanced Porous Bio-Hybrids with Inflammatory Targeting, ROS Scavenging, and Long-Term Drug Release for Ulcerative Colitis Treatment**

Luna Quan, Yang Ouyang, Weiwen Liang, Zixin Chen, Dongtian Miao, Bingna Zheng^*^, Dingcai Wu^*^, Rongkang Huang^*^

L. Quan, D. Miao, Prof. D. Wu

PCFM Lab, School of Chemistry,

Sun Yat-sen University, Guangzhou 510006, P. R. China

E-mail: wudc@mail.sysu.edu.cn

Y. Ouyang, W. Liang, Z. Chen, Dr. R. Huang

Colorectal Surgery Unit Ⅲ, Guangdong Institute of Gastroenterology, Biomedical Innovation Center, Guangdong Provincial Key Laboratory of Colorectal and Pelvic Floor Diseases, The Sixth Affiliated Hospital, Sun Yat-sen University, Guangzhou 510655, P. R. China

E-mail: huangrk3@mail.sysu.edu.cn

Dr. B. Zheng

The Eighth Affiliated Hospital, Sun Yat-sen University, Shenzhen 518000, P. R. China

E-mail: [zhengbn3@mail.sysu.edu.cn](mailto:zhengbn3@mail.sysu.edu.cn)

***Materials***

4-Vinylbenzyl chloride (VBC) was purchased from Shandong Xiya Chemical Co., Ltd. (China). Divinylbenzene (DVB), anhydrous ferric chloride (FeCl_3_), 1,2-dichloroethane (DCE), acrylamide (AM), 5-aminosalicylic acid (5-ASA), and N-hydroxysuccinimide (NHS) were purchased from Shanghai Aladdin Biochemical Technology Co., Ltd. (China). Sodium dodecyl sulfate (SDS), methanol, 5,5-dimethyl-1-pyrroline-N-oxide (DMPO), potassium persulfate (KPS), *N,N*-dimethylformamide (DMF), dimethyl sulfoxide (DMSO), 1-ethyl-3-(3-dimethylaminopropyl)carbodiimide (EDC), 2,2-diphenyl-1-picrylhydrazyl (DPPH), and Cy5.5-NHS ester were purchased from Shanghai Macklin Biochemical Co., Ltd. (China). Diethyl ether was purchased from Guangdong Guangshi Reagent Technology Co., Ltd (China). Phosphate‑buffered saline (PBS, with a concentration of 0.01 M and a pH of 7.4) was supplied by Wuhan Servicebio Technology Co., Ltd. (China). Luria Bertani (LB) broth was obtained from Guangdong Huankai Microbial Sci.&Tech. Co., Ltd. (China). *Escherichia coli* Nissle 1917 (EcN, ATCC 700928) and normal human colon mucosal epithelial (NCM460) cells were obtained from the Sixth Affiliated Hospital of Sun Yat-sen University (China). Fetal bovine serum (FBS), high glucose Dulbecco’s modified eagle medium (DMEM), and penicillin‑streptomycin (PS) were acquired from Gibco‑BRL Life Technologies, Inc. (USA). Cell counting kit‑8 (CCK‑8), calcein acetoxymethyl ester (Calcein AM)/propidium iodide (PI) double staining kit and Hoechst 33342 were procured from Beyotime Biotechnology Co., Ltd. (China). 2′,7′‐Dichlorodihydrofluorescein diacetate (DCFH-DA) was purchased from Beijing Solarbio Science & Technology Co., Ltd. (China). VBC and DVB were purified by passage through a basic alumina column, and other reagents were used without further purification.

***Synthesis of PCMS and xPCMS***

Poly(4-chloromethylstyrene) nanospheres with DVB pre-cross-linking (PCMS) were obtained by emulsion polymerization. 0.24 g of SDS dissolved in 40 mL of deionized (DI) water was added to a 100 mL three-necked round-bottom flask. 4 g of VBC and 0.10 g of DVB were added to the flask and pre-emulsified with stirring for 60 min under N_2_ atmosphere. 2 mL of KPS solution (0.041 g of KPS in 2 mL DI water) was added to the above mixture at 80 °C, and the emulsion polymerization was carried out for 6 h. The emulsion was broken by drop-wise addition into 100 mL of methanol under stirring, and then white solid was isolated by centrifugation (14500 rpm for 10 min), washed with DI water, methanol, and diethyl ether each for three times, and finally dried for 24 h at 60 °C. 2.5 g of PCMS was swollen in 40 mL of DCE under N_2_ for 3 h. 2.61 g of FeCl_3_ in 40 mL of DCE was then added to conduct the hyper-cross-linking at 80 °C for 18 h under stirring. The obtained product was filtered and washed with DI water, methanol, and diethyl ether each for three times, followed by Soxhlet extraction in methanol. The obtained solid was vacuum-dried at 60 °C for 24 h, producing microporous polymer nanospheres (*x*PCMS).^[1]^

***Synthesis of xPAM***

0.2 g of *x*PCMS, 20.4 mg of N',N'-bis[2-(dimethylamino)ethyl]-N,N-dimethylethane-1,2-diamine (Me_6_TREN, 0.089 mmol), 250.9 mg of AM (3.53 mmol), and 5 mL of DMF were placed in a Schlenk flask under N_2_ atmosphere. 12.7 mg of CuBr (0.089 mmol) was added to the flask. The flask was immersed in an oil bath at 35 ºC. After 12 h of polymerization, the reaction was stopped by exposure to air. The product was centrifuged (14500 rpm for 10 min), washed with diethyl ether and DI water each for three times, and then freeze-dried for 24 h.^[2]^

***Preparation of xPAM@ASA***

1 mg of *x*PAM and 1 mg of 5-ASA were dispersed in 1 mL of DMSO and stirred for 24 h. Solid was collected by filtration and washed with DMSO and DI water each for three times, to remove free 5-ASA. The obtained solid was freeze-dried for 24 h, yielding drug-loaded nanoparticles (*x*PAM@ASA).

***Preparation of E-xPAM and E-xPAM@ASA***

Prior to coupling reaction, *x*PAM were treated by plasma (150 W, 5 min). 5 × 10^10^ CFU of EcN was dispersed in 5 mL of DI water, followed by the addition of 5 mg of *x*PAM, 20 mg of EDC, and 15 mg of NHS into the EcN dispersion. The mixture was stirred for 3 h, and then the as-obtained probiotic-enhanced porous bio-hybrids (E-*x*PAM) were separated by centrifugation (4000 rpm, 5 min) and washed three times with DI water. E-*x*PAM@ASA were prepared using the same procedures as E-*x*PAM, with *x*PAM replaced by *x*PAM@ASA.^[3]^

***Material characterization***

Microstructures were investigated by scanning electron microscopy (SEM, S-4800, Hitachi, Japan) and transmission electron microscopy (TEM, JEOL JEM-2010HR, JEOL, Japan). Fourier-transform infrared (FTIR) spectra were recorded on an FTIR spectrometer (Tensor 27, Bruker, Germany). Thermogravimetric analysis (TGA) measurements were conducted on a thermogravimetric analyzer (TG 209F1 Iris, NETZSCH, Germany). Pore structure was investigated by an accelerated surface area and porosimetry system (ASAP 2020, Micromeritics, USA), and pore size distribution was calculated based on original density functional theory (DFT). Electron paramagnetic resonance (EPR) spectra were recorded on an EPR spectrometer (A300, Bruker, Germany). Ultraviolet‐visible (UV-vis) absorption spectra were recorded on a UV-vis spectrometer (Lambda 950, PerkinElmer, USA).

***In vitro drug release behavior***

To evaluate the drug release performance of *x*PAM@ASA, the anti-inflammatory drug 5-ASA was selected for in vitro drug release studies. The release behavior of 5-ASA was determined using a dialysis method. 2 mL of *x*PAM@ASA dispersion (2.5 mg mL^−1^) in PBS was placed into a dialysis bag and immersed in 8 mL of PBS at 37 °C with 120 rpm shaking. At predetermined time intervals, 3 mL of the release solution was withdrawn and replaced with an equal volume of fresh PBS. The UV-vis absorption spectra in the range of 240–300 nm were recorded to determine the release amount of 5-ASA over time.

***Reactive oxygen species (ROS) scavenging ability***

EPR experiments were carried out to confirm the •OH scavenging activities of *x*PAM and E-*x*PAM@ASA by using the TiO_2_/UV system to produce •OH. 300 μg of *x*PAM or 1.2 × 10^9^ CFU of E-*x*PAM@ASA was dispersed in 600 μL of TiO_2_ dispersion (5 mg mL^−1^), and then 50 μL of DMPO was added to the dispersion. After 5 min exposure to UV light (365 nm), EPR spectra were recorded.^[4]^

ROS scavenging ability of E-*x*PAM@ASA was further investigated by the scavenging assay of DPPH. 2 mL of DPPH ethanol solution (100 μM) was mixed with 4 × 10^9^ CFU of E-*x*PAM@ASA, and the mixture was incubated in the dark at 37 °C with shaking at 120 rpm for 1 h. 200 μL of the mixture was withdrawn and diluted. The UV-vis absorption spectra in the range of 450–600 nm were measured to monitor the changes in the DPPH signal.^[5]^

***Intracellular ROS detection***

A total of 5 × 10^3^ NCM460 cells were seeded in a 96-well plate for 24 h and then co-incubated with four groups: (1) untreated, (2) H_2_O_2_, (3) E-*x*PAM+H_2_O_2,_ and (4) E-*x*PAM@ASA+H_2_O_2_. The content of bio-hybrids was 4 × 10^8^ CFU mL^−1^ and the concentration of H_2_O_2_ was 400 μmol L^−1^. After 2 h of co-incubation, the cells were stained with diluted DCFH-DA and Hoechst 33342 for 60 min and 30 min, respectively. The green fluorescence indicating the intracellular ROS level and the blue fluorescence indicating the living cells were observed by a fluorescence microscope (DP80, Olympus, Japan).

***Flow cytometry***

A total of 2 × 10^5^ NCM460 cells were seeded in a 24-well plate for 24 h and then co-incubated with four groups: (1) untreated, (2) H_2_O_2_, (3) E-*x*PAM+H_2_O_2_, and (4) E-*x*PAM@ASA+H_2_O_2_. The content of bio-hybrids was 4 × 10^8^ CFU mL^−1^ and the concentration of H_2_O_2_ was 400 μmol L^−1^. After 2 h of co-incubation, cells were washed twice with PBS and stained with diluted DCFH-DA for 30 min. Following trypsinization and filtration through a nylon mesh, cells were resuspended in PBS and analyzed using a flow cytometer (CytoFLEX S, Beckman Coulter, USA). Data were processed with FlowJo v10.8 (BD biosciences, USA), and mean fluorescence intensity was quantified to evaluate intracellular ROS levels.

***Probiotic proliferation activity assay***

To evaluate the proliferation activities of EcN and E-*x*PAM@ASA, each dispersion of EcN and E-*x*PAM@ASA was diluted at a 1:100 ratio in LB medium and incubated at 37 °C with shaking (120 rpm) for 8 h. After incubation, the cultures were serially diluted and plated onto LB agar plates. The plates were then incubated at 37 °C for 15 h, and the colonies were counted using the plate counting method to assess probiotic growth.

***Probiotic motility evaluation***

To evaluate the motilities of EcN and E-*x*PAM@ASA, 10 μL of each dispersion was placed onto a glass slide, and a coverslip was carefully applied to prevent evaporation while allowing probiotic movement. The motilities of probiotic and bio-hybrids were observed and recorded under an inverted microscope (DP80, Olympus, Japan). The trajectories were analyzed by converting the recorded frames into images using the manual tracking plug-in of ImageJ software (NIH, USA). The average motility speeds in different groups were determined by calculating the distance traveled over time.

***In vitro cytocompatibility test***

Initially, NCM460 cells were seeded into a 96-well plate at a density of 5 × 10^3^ cells per well, with each well receiving 100 µL of complete DMEM medium (DMEM medium supplemented with 10% FBS and 1% PS). The cells were incubated in a humid environment with 5% CO_2_ at 37 °C for 24 h. In the control group, cells were treated with the complete medium. In the *x*PAM group, cells were treated with the complete medium and *x*PAM (0.1 mg mL^−1^). After 1, 2, and 3 days, the liquid was carefully removed, and the wells were washed three times with PBS. Cells were incubated with a solution of Calcein AM (2 µM) and PI (8 µM) for 30 min and observed under a fluorescence microscope (DP80, Olympus, Japan). Meanwhile, the CCK-8 assay was carried out to determine the proliferation of cells.

***In vivo targeting performance*** ***assessment***

To evaluate the targeting of E-*x*PAM in colitis mice, an in vivo imaging system (IVIS) was employed. Cy5.5-NHS ester was used to label E-*x*PAM for fluorescence tracking. Healthy mice were fed with normal drinking water without any treatment. The DSS-induced mice received 3% DSS in drinking water to induce the ulcerative colitis (UC) model. After 4 days, both the healthy and DSS-induced mice were administered by gavage with equal amounts of Cy5.5-labeled E-*x*PAM. Mice were euthanized, and their intestines were collected for fluorescence imaging using IVIS on the 5th day. The images of the tissues were recorded and analyzed using Living Image 4.2 software (PerkinElmer, USA).

***In vivo retention performance*** ***assessment***

To evaluate the retention of *x*PAM and E-*x*PAM in colitis mice. The DSS-induced mice received 3% DSS in drinking water for 7 days to induce the UC model, followed by normal water. DSS-induced mice were divided into *x*PAM and E-*x*PAM groups and administered by gavage with equal amounts of Cy5.5-labeled *x*PAM and Cy5.5-labeled E-*x*PAM on the fourth day, respectively. Fluorescence signals were tracked on Day 0 (6 h post gavage), 1, 3 and 7 using IVIS. On Day 8, the mice were euthanized, and their intestines were collected for fluorescence imaging. The images of the mice and tissues were recorded and analyzed using Living Image 4.2 software (PerkinElmer, USA).

***UC murine model***

Female C57BL/6 mice aged 8-10 weeks were randomly divided into four groups. The healthy group was fed with normal water without any treatment. The other three groups received 3% DSS in drinking water for 7 days to induce UC model, followed by normal water. Starting from Day 4, the control, *x*PAM@ASA, and E-*x*PAM@ASA groups were administered 100 μL of PBS, *x*PAM@ASA dispersion (1 mg mL^−1^), and E-*x*PAM@ASA dispersion (4 × 10^9^ CFU mL^−1^), respectively, via gavage once daily. The treatment continued until Day 10. During the experiment, mice were weighed daily. Body weight loss, stool characteristics, and hematochezia were assessed and scored based on the disease assessment criteria shown in Table S1. Disease activity index (DAI) was calculated as the average of all individual scores. The body weight loss was calculated as follows:

$$Body weight loss \left( \% \right)=\frac{Weight on Day 0-Weight on Day n}{Weight on Day 0}\times100$$

After 10 days, all the mice were sacrificed in accordance with the provisions of the Animal Welfare Act. The entire colon was excised, leaving a 3 mm segment of the small intestine as the colonic starting point. The colon was then photographed, and its length was measured. Subsequently, each colon tissue was rolled into a Swiss roll and collected for further analysis.

***Histological examination and immunohistochemistry staining***

The collected colon tissues were immediately fixed in 4% PFA solution at room temperature for 24 h, and then sent to Wuhan Servicebio Technology Co., Ltd. (China) for staining. Histological evaluations using hematoxylin-eosin (HE) staining and Alcian Blue (AB) staining were conducted to assess the pathological condition, mucin secretion, and tissue recovery in colonic tissues after treatment. Immunohistochemistry staining of IL‑6 and TNF-α was performed to assess the inflammatory state of the tissues.

***In vivo toxicity evaluation***

In vivo toxicities of *x*PAM@ASA and E-*x*PAM@ASA were evaluated through pathological analysis of major organs in mice. After 10 days, the mice were sacrificed, and the heart, liver, spleen, lung, and kidney sections were subjected to HE staining.

***Microbiota 16S ribosomal RNA gene sequencing***

16S ribosomal RNA (16S rRNA) gene sequencing and analysis were conducted at Majorbio Bio-Pharm Technology Co. Ltd. (China). First, total bacterial DNA was extracted from feces samples of mice after various treatments. Hypervariable V3-V4 regions of the bacterial 16S rRNA gene were amplified with primer pairs 338F (5'-ACTCCTACGGGAGGCAGCAG-3') and 806R (5'-GGACTACHVGGGTWTCTAAT-3'), and the obtained sequences were detected on the Illumina NextSeq 2000 PE300 platform. Data analysis was performed. α diversity was analyzed to examine the richness and diversity of species in individual sample with Chao and Shannon index, while β-diversity was analyzed with principal component analysis. Moreover, the species classification was obtained based on the sequence composition of the amplicon sequence variant. Microbial composition was analyzed on phylum and genus levels.

***Animal ethics statement***

Experimental mice were purchased from and bred at the Animal Center of Sun Yat-sen University (China). Animal experiments were reviewed and approved by the Institutional Animal Care and Use Committee (IACUC) at Sun Yat-Sen University (China, license number: IACUC-2024001304).

***Statistical analysis***

Statistical analyses were performed using Origin 2025 software (Origin Lab Incorporation, Northampton, USA). Data were expressed as mean ± standard deviation (SD). Statistical differences between groups were determined by one‑way analysis of variance (ANOVA). Statistical significance indicated as **p* < 0.05, ***p* < 0.01, and ****p* < 0.001.


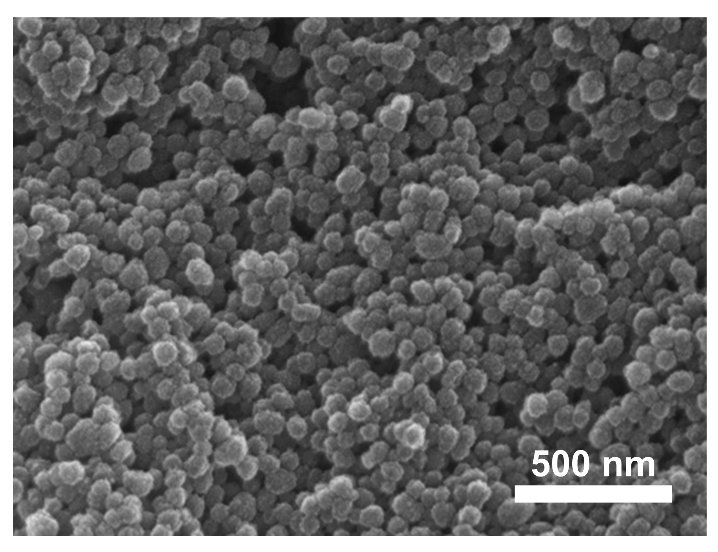


**Figure S1.** SEM image of *x*PCMS.


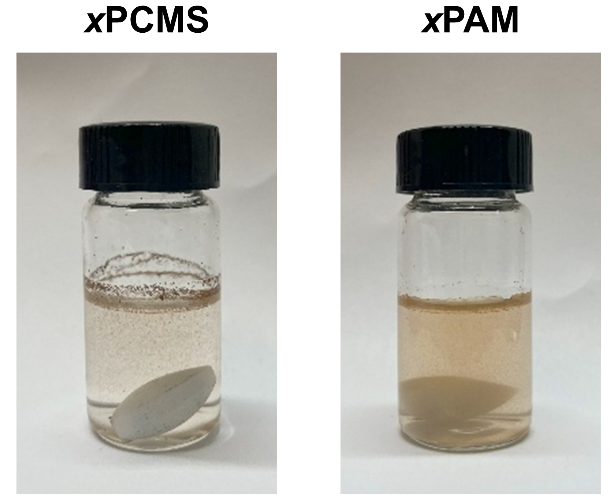


**Figure S2.** Digital photos of *x*PCMS and *x*PAM dispersed in water.


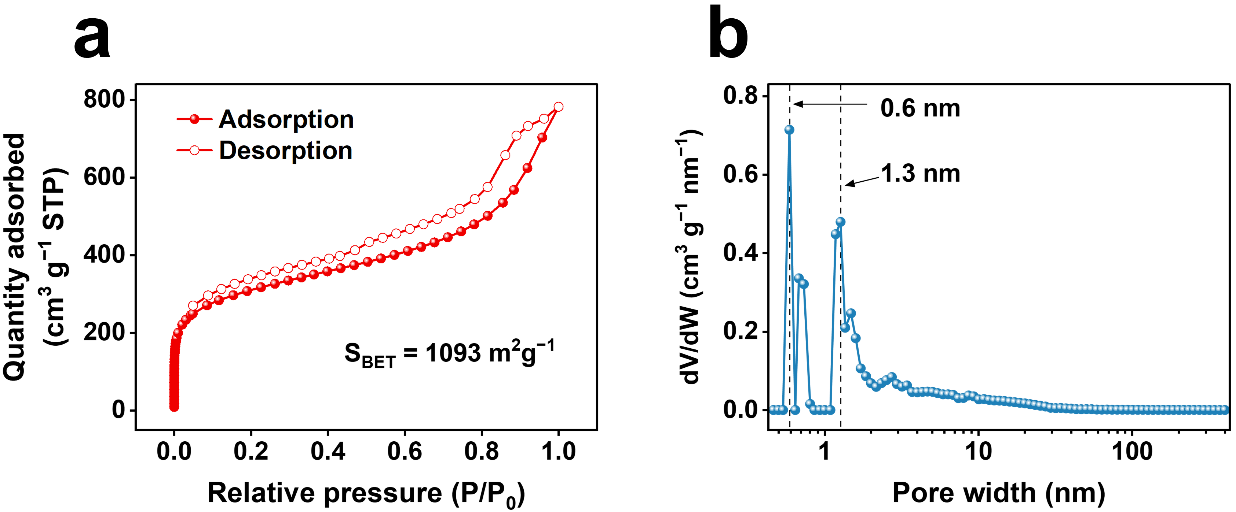


**Figure S3.** N_2_ adsorption-desorption isotherm (a) and DFT pore size distribution curve (b) for *x*PCMS.


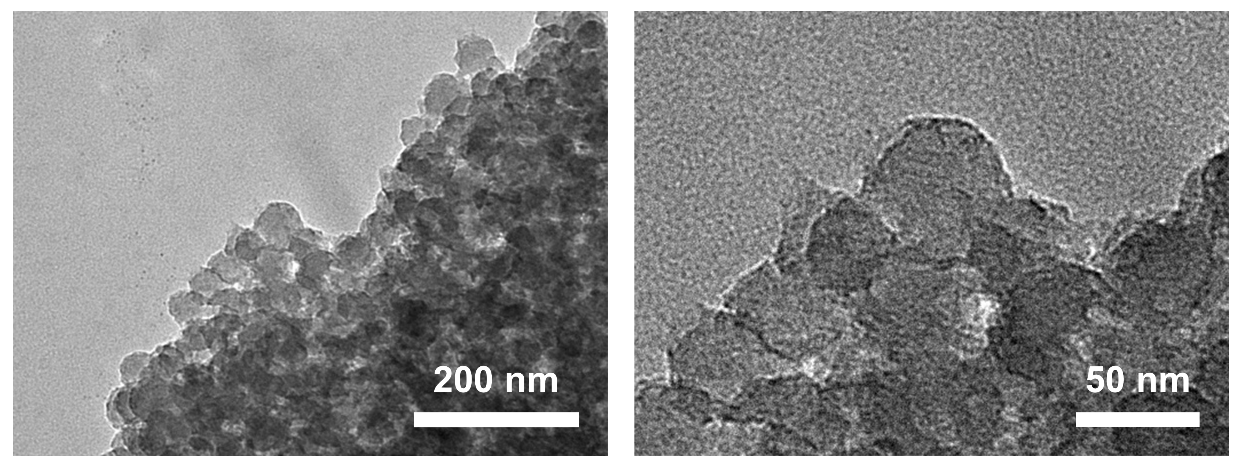


**Figure S4.** TEM images of *x*PAM.


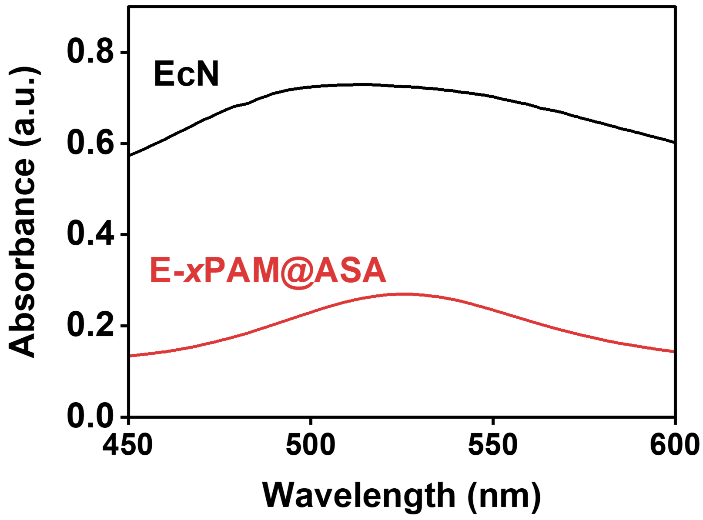


**Figure S5.** UV-vis absorption curves of DPPH in the presence of EcN and E-*x*PAM@ASA.


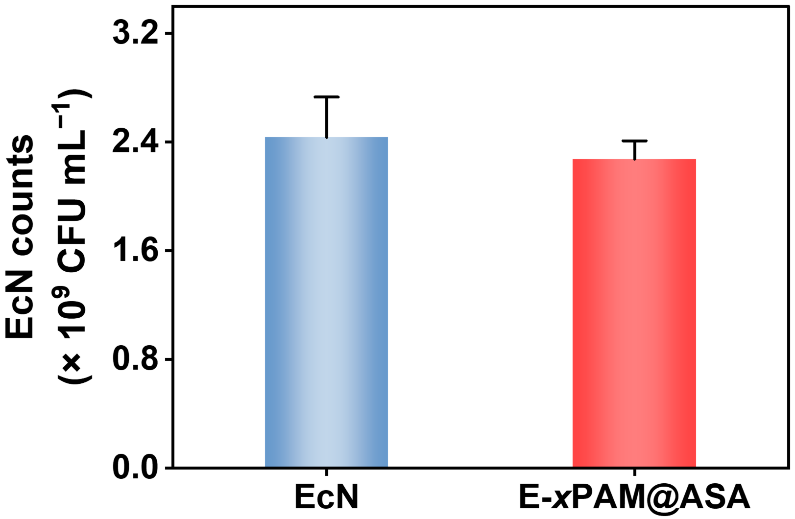


**Figure S6.** Colony count statistics of EcN and E-*x*PAM@ASA after coupling reaction. The data are the mean ± SD (n = 3 independent samples).


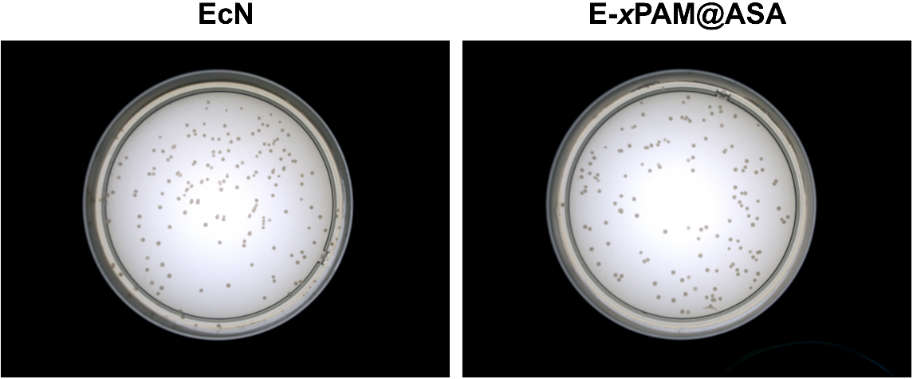


**Figure S7.** Colonies of EcN and E-*x*PAM@ASA on LB agar plates after 8 h incubation in LB medium.


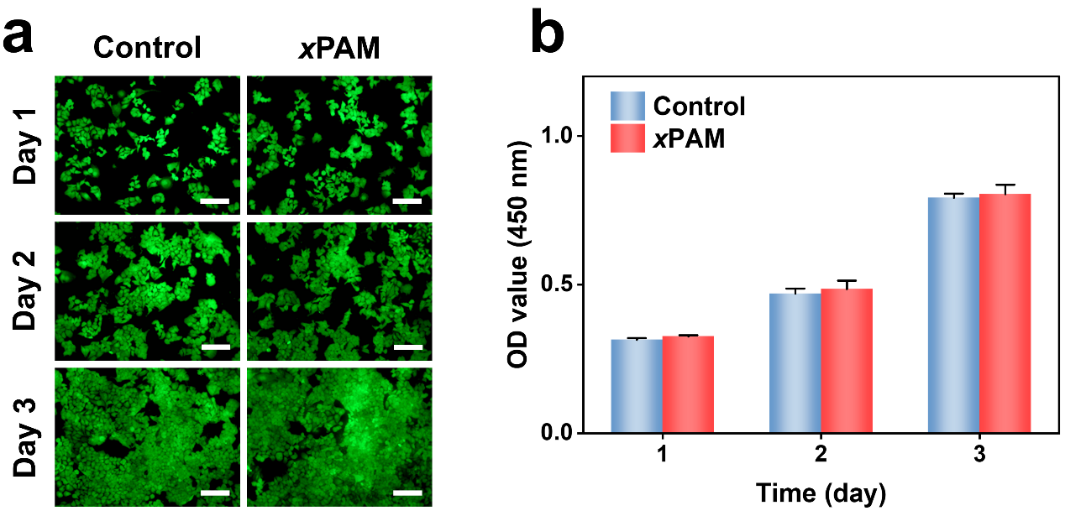


**Figure S8.** a) Fluorescence images and b) CCK-8 assay of NCM460 cells cultured for 1, 2, and 3 days in complete DMEM medium and *x*PAM-conditioned media. Scale bars: 50 μm (a). The data are the mean ± SD (n = 3 independent samples).


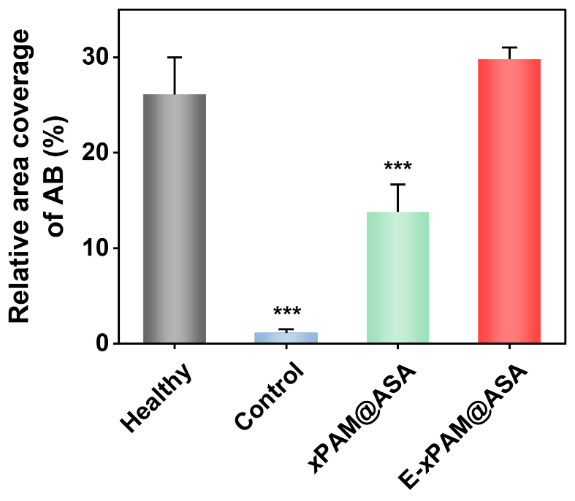


**Figure S9.** Quantitative analysis of AB staining. The data are the mean ± SD (n = 3 independent samples; ****p* < 0.001).


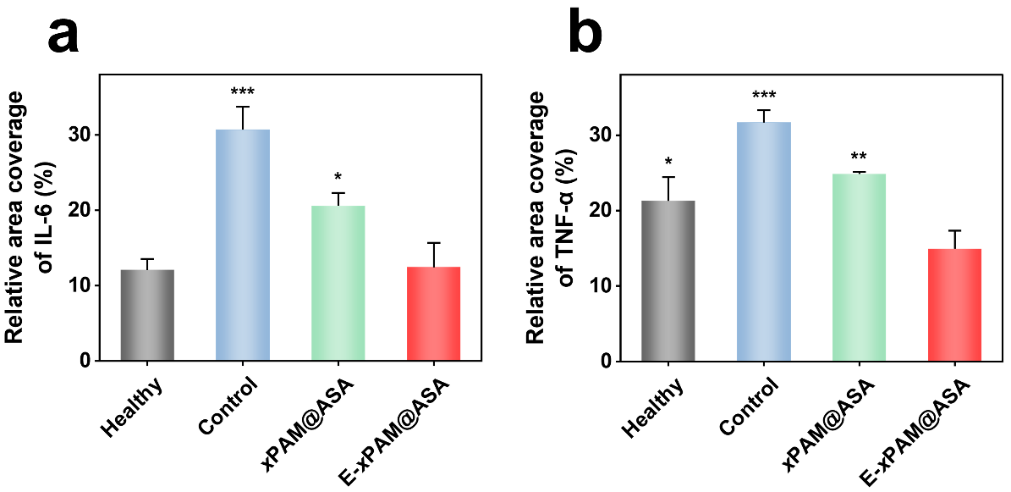


**Figure S10.** Quantitative analysis of a) IL-6 and b) TNF-α staining. The data are the mean ± SD (n = 3 independent samples; **p* < 0.05, ***p* < 0.01, ****p* < 0.001).


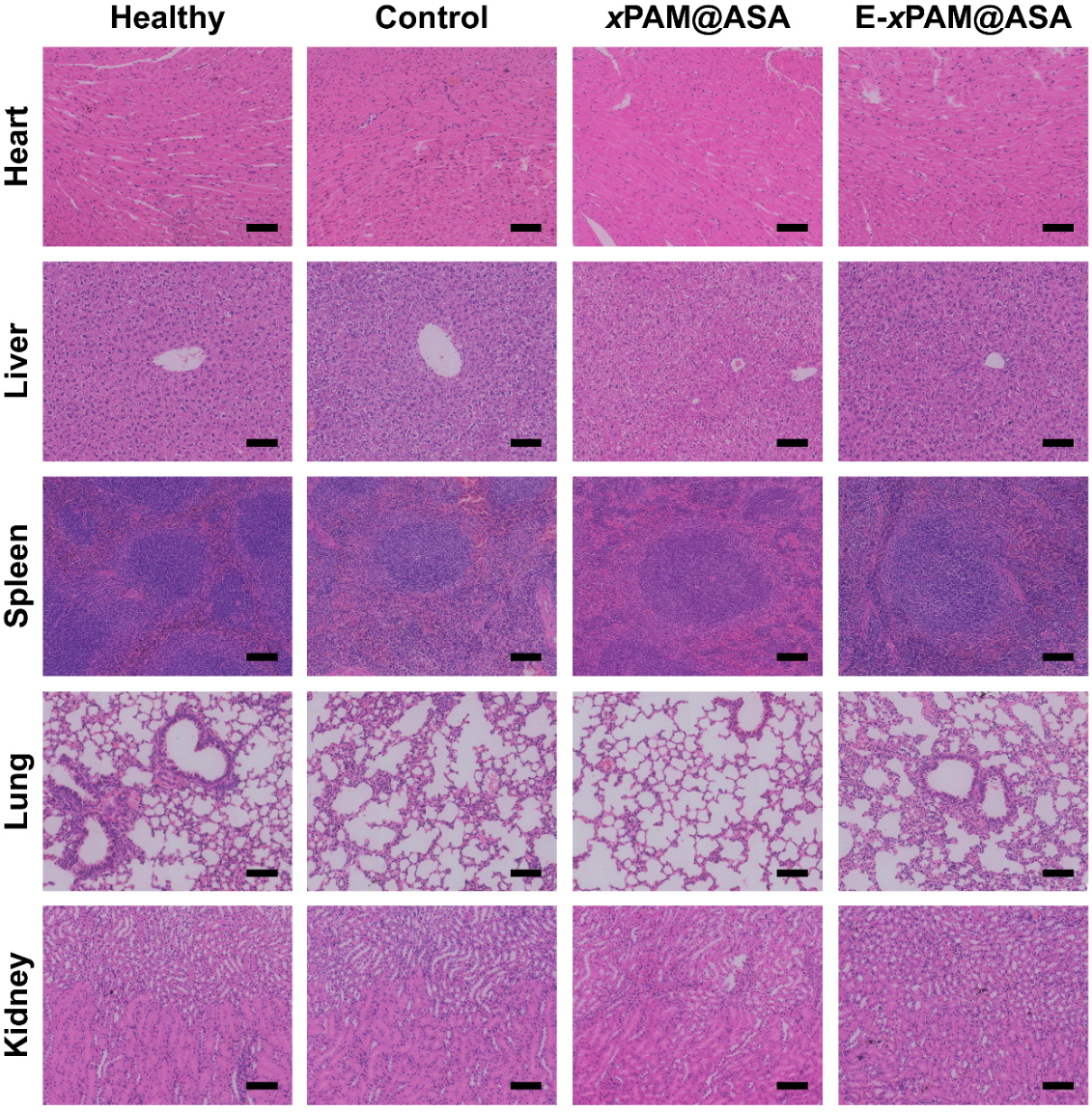


**Figure S11.** HE staining images of the heart, liver, spleen, lung, and kidney in each group. Scale bars: 100 μm.


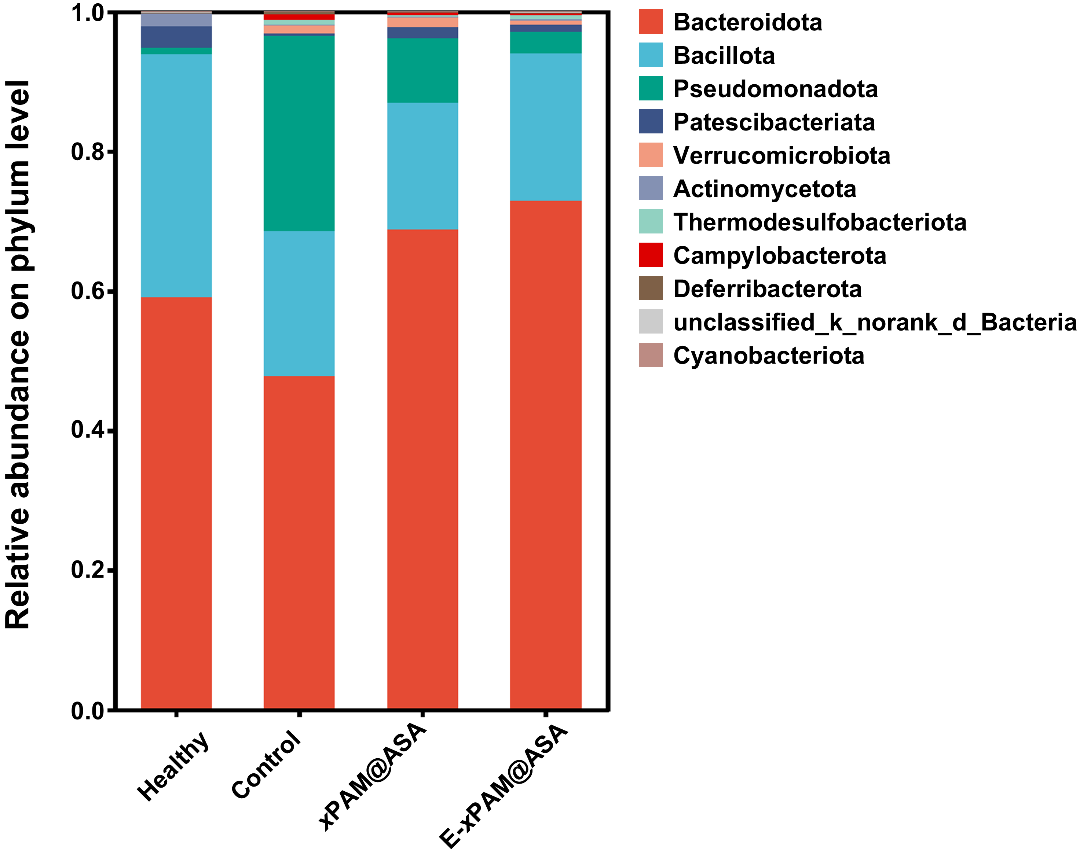


**Figure S12.** Relative abundance of fecal microbiome on phylum level.


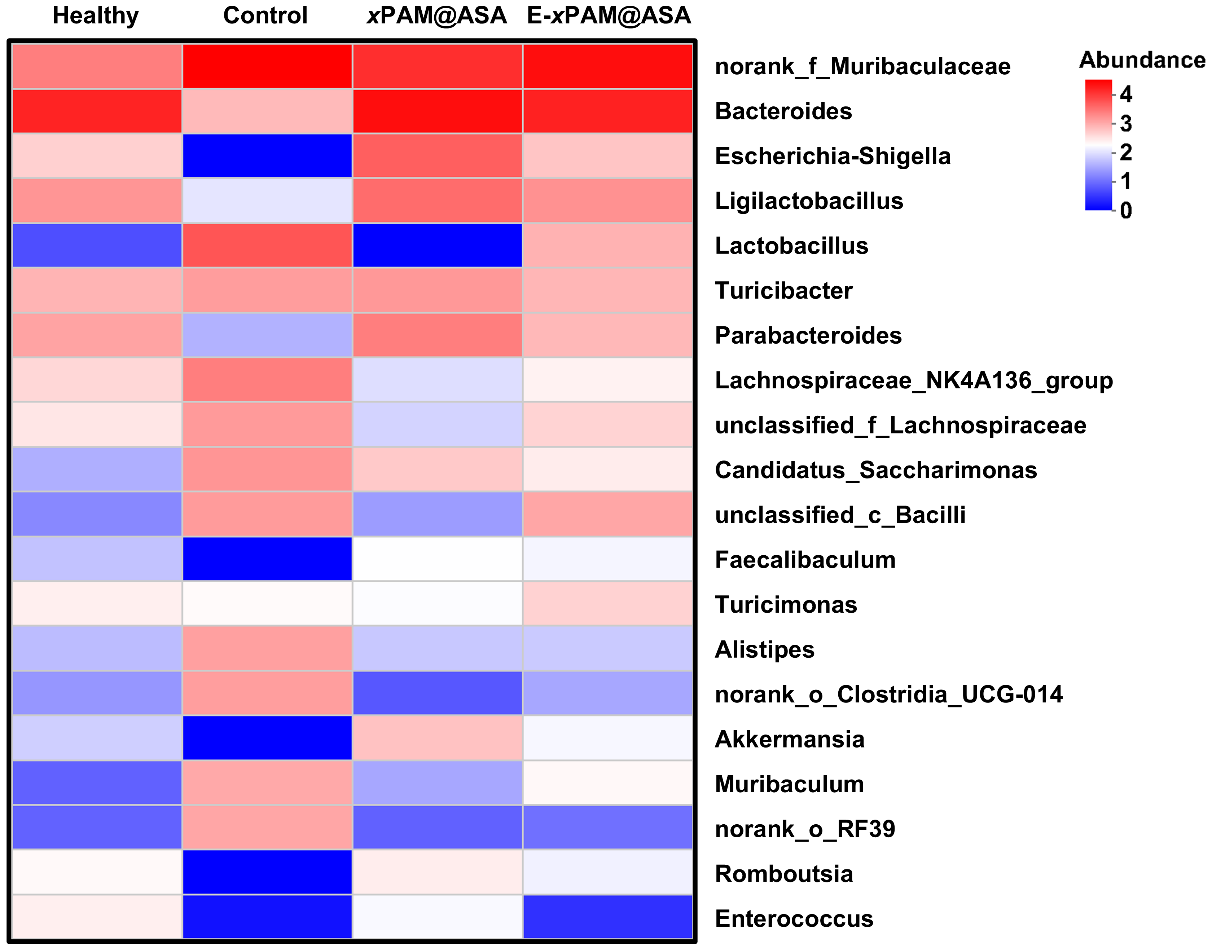


**Figure S13.** Heatmap depicting the relative abundance of fecal microbiome on genus level.

**Table S1.** DAI Scoring Criteria

| Score | Weight loss (%) | Stool characteristics | Hematochezia |
| --- | --- | --- | --- |
| 0 | ≤ 0 | Normal stool | Normal |
| 1 | 0 - 5 | Loose stool | Fecal occult blood |
| 2 | 5 - 10 |  |  |
| 3 | 10 - 15 | Watery stool | Gross blood |
| 4 | > 15 |  |  |

# *References*

[1] B. Li, X. Huang, L. Liang, B. Tan, Synthesis of Uniform Microporous Polymer Nanoparticles and Their Applications for Hydrogen Storage. *J. Mater. Chem.* **2010,** *20*, 7444.

[2] W. Mai, B. Sun, L. Chen, F. Xu, H. Liu, Y. Liang, R. Fu, D. Wu, K. Matyjaszewski, Water-Dispersible, Responsive, and Carbonizable Hairy Microporous Polymeric Nanospheres. *J. Am. Chem. Soc.* **2015,** *137*, 13256-9.

[3] J. X. Fan, M. Y. Peng, H. Wang, H. R. Zheng, Z. L. Liu, C. X. Li, X. N. Wang, X. H. Liu, S. X. Cheng, X. Z. Zhang, Engineered Bacterial Bioreactor for Tumor Therapy Via Fenton-Like Reaction with Localized H_2_O_2_ Generation. *Adv. Mater.* **2019,** *31*, e1808278.

[4] F. Cao, L. Jin, Y. Gao, Y. Ding, H. Wen, Z. Qian, C. Zhang, L. Hong, H. Yang, J. Zhang, Z. Tong, W. Wang, X. Chen, Z. Mao, Artificial-Enzymes-Armed Bifidobacterium Longum Probiotics for Alleviating Intestinal Inflammation and Microbiota Dysbiosis. *Nat. Nanotechnol.* **2023,** *18*, 617-27.

[5] W. He, Q. Li, M. Zhang, Y. Qin, S. Luo, Z. Cen, Y. Li, B. Zheng, S. Wu, D. Wu, Leaf-Mimicking Superstructured Porous Membrane with Multiple Bioactive Pro-Healing Functions for Effective Guided Bone Regeneration. *Adv. Funct. Mater.* **2024,** *34*, 2316679.
